# Ureametry

**Published:** 1890-07-19

**Authors:** 


### UREAMETRY.

Dr. Cruise, of Dublin, in a note on ureametry, describes the instrument he uses. It consists of two parts. First, a vertical glass tube, closed at the top, and bent sharply below where it expands into a bulb, having an orifice at the upper part; secondly, a pipette with an elastic rubber nipple. Both parts are graduated, the vertical tube so as to show the quantity of urea (as indicated by the volume of nitrogen) in each cubic centimetre of urine tested, while the pipette is graduated so as to show one cubic centimetre. First the vertical tube is filled with a solution of hypobromite of sodium. Holding it vertically, the operator pours the solution into the bulb. When rather more than half full, the apparatus is inclined horizontally till the entire tube is filled, and a little left in the bulb—about one-third. Then it is restored to a vertical position. Secondly, the operator draws into the pipette, by means of the elastic nipple, a cubic centimetre of the urine to be tested. Finally he passes the nozzle of the pipette into the bulb, until the point is exactly under the vertical tube, and slowly compresses the nipple. The urine being lighter than the hypobromite solution rises through it, and on its way the contained urea is decomposed and its nitrogen set free. This gas collects at the upper end of the vertical tube. Being an exact measure of the urea from which it is derived, it enables the operator to ascertain the exact quantity of urea in the cubic centimetre of urine. The graduation is so arranged as to indicate the proportion of urea per litre of urine. As the metrical system of measurement is not familiar to all practitioners, Messrs. Southall, of Birmingham, add a scale graduated for the quantity of urea per ounce. Dr. Cruise observes that if physicians would use this instrument habitually in the numbers of cases of renal deficiency which are met with, they would find it very valuable. An easy method of determining the quantity of urea in the urine has long been a desideratum. The above appears to be a simplification of Russell and West's method, which is based on the fact that hypobromous acid decomposes urea into water, carbonic acid, and nitrogen. For accurate determination Liebeg's method has been generally in use, which consists of a somewhat complicated volumetric analysis. So far as we can judge, without having actually experimented, Dr. Cruise's method seems worthy of extended trial.

